# Beyond the *PEL* surveyance: Genome editing of the *OsPEL* family enhances photosynthesis efficiency in rice

**DOI:** 10.1093/plcell/koaf247

**Published:** 2025-10-15

**Authors:** Christian Damian Lorenzo

**Affiliations:** Assistant Features Editor, The Plant Cell, American Society of Plant Biologists; Center for Plant Systems Biology, VIB, Gent 9052, Belgium; Department of Plant Biotechnology and Bioinformatics, Ghent University, Gent 9052, Belgium

Photosynthesis is a major target of improvement strategies to boost crop productivity. One strategy focuses on engineering the C4 metabolic pathway, or aspects of it, into C3 crops such as rice (Croce et al. 2024). C4 plants have a specialized photosynthetic metabolism in which CO_2_ is first incorporated into a reduced C4 acid in mesophyll chloroplasts. The C4 acid then moves to the bundle sheath chloroplasts, where it concentrates CO_2_ around Rubisco, the key enzyme involved in CO_2_ fixation. This optimizes carbon fixation by reducing photosynthetic losses due to photorespiration, a process where Rubisco uses O_2_ instead of CO_2_ as its substrate (Mukundan et al. 2024). Unfortunately, engineering the C4 pathway into C3 plants is complex. Overexpression of the transcription factor *GOLDEN2-LIKE* (*GLK*) from a C4 crop (maize) in rice (*Oryza sativa*) results in a pseudo-C4 anatomy with increased chloroplast development (Choi et al. 2024). However, similar approaches modifying the *GLK* from rice itself had little effect, hinting at a more complex regulation in this crop. Identification of other potential genes to improve photosynthetic efficiency has highlighted the *PSEUDO-ETIOLATION IN LIGHT* (*PEL*) family as a promising hub. *PEL* genes code for microproteins that act as negative regulators of photosynthesis. *PEL* knockout (KO) mutants exhibit higher chlorophyll levels and increased biomass as compared with wild type (wt) plants (Kim et al. 2023). Mechanistic studies indicate that PEL proteins act through inhibition of the DNA-binding properties of transcription factors (Kim et al. 2023).

Delving deeper into the potential role of PELs as an avenue for photosynthesis improvements, Heebak Choi and collaborators (2025) performed a functional analysis of the PELs in rice. The researchers identified 3 orthologs of *Arabidopsis thaliana* PELs: *O. sativa PEL1* (*OsPEL1*), *OsPEL2*, and *OsPEL3*. Tissue expression studies showed that all *OsPEL*s were highly expressed in young shoots, particularly in mesophyll cells, the main site of carbon fixation in C3 plants. Phenotypical characterization of *OsPEL* overexpression (OE) lines (OsPEL1OE) revealed pale-green coloration in several tissues and reductions of chlorophyll levels and biomass as compared with wt plants. In generated KO lines, a single *OsPEL* KO did not cause a distinct phenotype, while triple-KO mutants (*Ospel1,2,3*) exhibited dark green leaves with increases in chlorophyll levels up to 80%. They also had more developed grana stacks (an increased number of thylakoid membrane layers), higher efficiency of CO_2_ assimilation, and greater plant biomass and grain weight.

The group screened for potential OsPEL protein–protein interactors through a yeast 2-hybrid assay. Among others, they identified OsGLK1, an orthologue to GLK in *Arabidopsis*, as well as PHOTOSYSTEM I ASSEMBLY 2 (PSA2), a key player in photosystem I development. Both proteins interacted with all 3 OsPELs through a conserved domain of 37 to 39 amino acids. Considering the previous evidence of OsPEL inhibiting DNA binding, the researchers analyzed the subcellular localization and accumulation of OsPEL, OsGLK1, and OsPSA2 in wt, OE, and KO lines. Isolated OsPEL1 and OsGLK1 were localized in the cytoplasm and nucleus, while OsPSA2 was localized in chloroplasts in wt plants. Nevertheless, assays performed in *Ospel1,2,3* and the OsPEL1OE revealed that OsGLK1 nuclear distribution increased in the former and decreased in the latter. Such effects were not observed when expressing a *OsGLK1* lacking the 37 amino acids needed for interaction. Likewise, PSA2 chloroplast localization was severely disrupted in OsPEL1OE lines. These findings suggest that OsPEL1 acts by sequestering OsGLK1 in the cytoplasm and reducing PSA2 subcellular distribution.

Finally, to better understand the action of *OsPEL*s at the transcriptomic level, bulk RNA sequencing was carried out on OE and KO lines. Interestingly, dark green *Ospel1,2,3* plants (due to the unrestricted localization of GLKs) displayed high *OsPEL* transcript levels, suggesting negative feedback regulation of expression within the OsPEL family, as well as other photosynthesis-associated nuclear genes. Accordingly, pale-green OE lines (caused by the cytoplasmic sequestration of GLKs and PSA2 by OsPELs) showed opposite trends. Altogether, the information points toward a complex regulatory pathway in rice where *PEL*s act as surveyors, fine-tuning chloroplast homeostasis at the transcriptional and protein levels (Fig.). These results reveal insights into the complex regulation mediated by OsPEL genes in rice and how this could be employed to improve photosynthesis.

**Figure.**
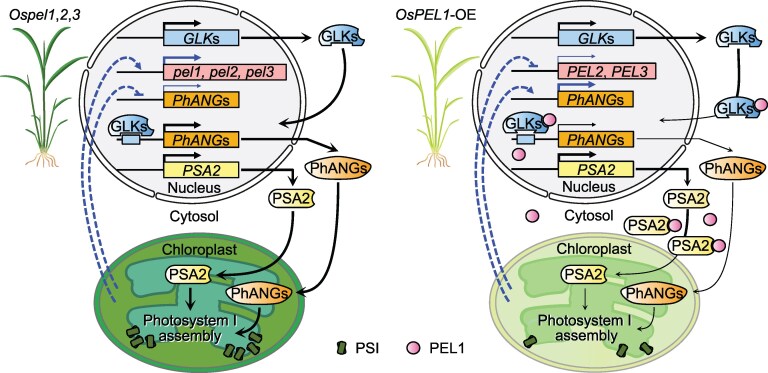
Model depicting the complex regulatory mechanism of chloroplast homeostasis in rice mediated by the OsPEL-GLK-PSA2 hub. Adapted from Figure 9 of Choi et al. (2025).

## Recent related articles in *The Plant Cell*

- Pei and collaborators (2025) showed that the transcription factor DEAR1 plays a role regulating chlorophyll biosynthesis and degradation in tomato.
- Croce and collaborators (2024) performed a case-detailed perspective review on the latest advancements aimed at optimizing photosynthetic efficiency, focusing on potential avenues for improvements and bottlenecks.

## Data Availability

Not applicable.

## Dive Curated Terms

The following phenotypic, genotypic, and functional terms are of significance to the work described in this paper:

PSA2 Gramene: AT2G34860
PSA2 Araport: AT2G34860
